# Performance Analysis of Non-Interferometry Based Surface Plasmon Resonance Microscopes

**DOI:** 10.3390/s21155230

**Published:** 2021-08-02

**Authors:** Sorawit Tontarawongsa, Sarinporn Visitsattapongse, Suejit Pechprasarn

**Affiliations:** 1 Department of Biomedical Engineering, School of Engineering, King Mongkut’s Institute of Technology, Ladkrabang, Bangkok 10520, Thailand; 62601082@kmitl.ac.th (S.T.); sarinporn.vi@kmitl.ac.th (S.V.); 2 College of Biomedical Engineering, Rangsit University, Pathum Thani 12000, Thailand

**Keywords:** surface plasmon microscopy, surface plasmon resonance, quantitative measurement, instrumentation

## Abstract

Surface plasmon microscopy has been of interest to the science and engineering community and has been utilized in broad aspects of applications and studies, including biochemical sensing and biomolecular binding kinetics. The benefits of surface plasmon microscopy include label-free detection, high sensitivity, and quantitative measurements. Here, a theoretical framework to analyze and compare several non-interferometric surface plasmon microscopes is proposed. The scope of the study is to (1) identify the strengths and weaknesses in each surface plasmon microscopes reported in the literature; (2) quantify their performance in terms of spatial imaging resolution, imaging contrast, sensitivity, and measurement accuracy for quantitative and non-quantitative imaging modes of the microscopes. Six types of non-interferometric microscopes were included in this study: annulus aperture scanning, half annulus aperture scanning, single-point scanning, double-point scanning, single-point scanning, at 45 degrees azimuthal angle, and double-point scanning at 45 degrees azimuthal angle. For non-quantitative imaging, there is a substantial tradeoff between the image contrast and the spatial resolution. For the quantitative imaging, the half annulus aperture provided the highest sensitivity of 127.058 rad/μm^2^ RIU^−1^, followed by the full annulus aperture of 126.318 rad/μm^2^ RIU^−1^. There is a clear tradeoff between spatial resolution and sensitivity. The annulus aperture and half annulus aperture had an optimal resolution, sensitivity, and crosstalk compared to the other non-interferometric surface plasmon resonance microscopes. The resolution depends strongly on the propagation length of the surface plasmons rather than the numerical aperture of the objective lens. For imaging and sensing purposes, the recommended microfluidic channel size and protein stamping size for surface plasmon resonance experiments is at least 25 μm for accurate plasmonic measurements.

## 1. Introduction

**Figure 1:**
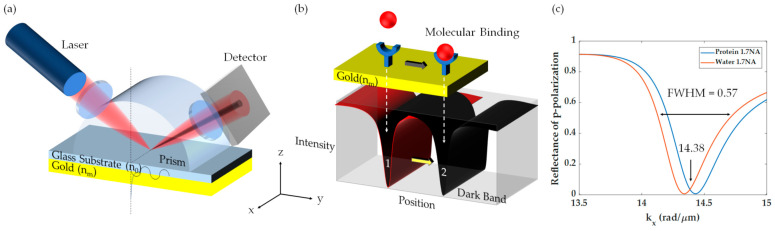
(**a**) Kretschmann configuration, (**b**) molecular binding and SPR reflectance spectra, and (**c**) SPR dips for bare gold 50 nm with water backing and the 10 nm uniform protein layer with a BSA protein refractive index of 1.4.

Surface plasmon resonance (SPR) is a guided electromagnetic wave effect on a noble metal surface, in which light is coupled to a surface wave mode through light-matter interaction [1]. The SPR has been utilized in a broad range of biomedical applications, for example, protein kinetics binding [2,3,4], immunoassay [5,6], refractive index sensing [7,8], and voltage sensing [9,10], and ultrasonic sensing [11]. For biomedical applications, the SPR can be excited in an optical configuration called Kretschmann configuration [12], which comprises a *p*-polarized coherent light source, a high refractive index prism, and a plasmonic thin-film as shown in Figure 1a. The light momentum at the plasmonic resonant condition can couple to the SPR effect and appear as a dark band in reflectance spectra due to the coupling process [13], and the minimum intensity position in the reflectance dip is called plasmonic angle, θ_sp_. In addition, the SPR is sensitive to the local refractive index change on the surface of the plasmonic metal resulting in a shift of the SPR reflectance dip position as shown in Figure 1b,c.

Yeatman and Ash proposed the first microscope configuration combining the SPR with the optical microscope in 1987 [14], in which the surface plasmons (SP) were excited at an oblique angle through a prism. One of the key findings is a tradeoff between spatial resolution and SPR sensitivity. Due to the leaky wave nature and attenuation of the SP [1], the plasmon propagation length degrades the spatial resolution of SPR optical microscopes [15]. The SP excited at one position travels several microns away from its excitation position as depicted in Figure 1a leading to a poor resolution.

Thanks to the development in high numerical aperture (NA) objective lenses, they have made SP excitation more convenient under a conventional optical microscope configuration [16,17]. Several SPR microscope configurations [18,19] can achieve good sensitivity without compromising the spatial resolution. The SPR microscopes have demonstrated their capability in microscopic scale biosensing applications, such as recently, an SPR microscope has been employed to measure the binding kinetics of spike protein in SARS CoV-2 or COVID-19 virus with angiotensin-converting enzyme 2 (ACE2) [20]. A research group in France has imaged and located a single 10–200 nm nanoparticle using a heterodyne *V(z)* SPR interferometric microscope [21]. Scientists have also employed the SPR microscope in surface-enhanced Raman microscopy and achieved single-molecule sensitivity [22,23].

Several SPR microscopes have been developed and reported in the literature [24,25,26]. However, there is no direct performance comparison for the microscopes. Therefore, here a theoretical framework to analyze and compare different SPR microscopes’ performance is proposed. Non-quantitative imaging microscopes and quantitative imaging microscopes are investigated and discussed. The non-quantitative imaging mode is that the microscopes provide images without recovering plasmonic angles corresponding to each position of the image; meanwhile, the quantitative imaging mode measures the plasmonic angles for each position of the image. To the best of the authors’ knowledge, this has never been reported before in the literature.

## 2. Materials and Methods

### 2.1. Optical Response Simulation Using Rigorous Coupled-Wave Theory

**Figure 2:**
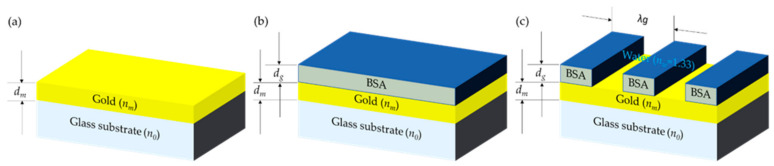
Schematic diagram of simulated structures, (**a**) bare gold sample in water backing environment, (**b**) uniform BSA protein-coated sample, and (**c**) 50 to 50% fill-factor grating sample.

A structured surface is needed to compute the spatial resolution of a microscope. A one-dimensional grating comprises 10 nm thick (*d_g_*) bovine serum albumin (BSA) protein stripes with the protein refractive index *n_g_* of 1.4 coated on a uniform gold-coated surface with the refractive index of gold of 0.1834 + 3.4332i [27] and the thickness *d_m_* of 50 nm, as depicted in Figure 2. There are three types of the sample included in this study, which are (1) a uniform gold sensor with the complex refractive index of *n_m_* and the thickness of *d_m_* surrounded by water ambient with the water refractive index *n_w_* of 1.33, (2) a uniform protein coated on the gold sensor with the BSA protein refractive index of 1.4 and the thickness of 10 nm, and (3) a 50 to 50% fill-factor protein grating sample with the grating period of *λ_g_* and the thickness of the stripes *h_g_*.

**Figure 3:**
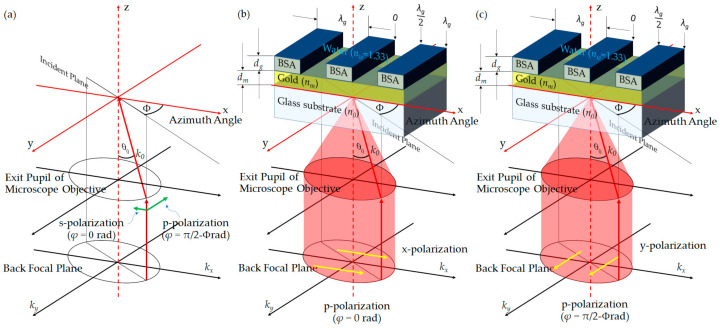
Shows (**a**) optical incidence and polarization direction, (**b**) x-polarization in the BFP, and (**c**) y-polarization in the BFP.

Rigorous coupled-wave theory [28,29] was employed to calculate the reflected magnetic field for *p*-polarization and the reflected electric field for s-polarization for a conical mount problem. The software has been developed in-house under MATLAB2019a utilizing parallel computing and graphic processing unit computing. A coherent light source with the free space wavelength *λ* of 633 nm illuminates the samples with the incident direction defined using the plane of incidence *ϕ* (azimuthal angle), incident angle *θ*_0_, and the polarization angle *φ* as shown in Figure 3. All the simulation results reported here were simulated with sufficient diffracted orders of 271 to ensure that the simulation convergence has been achieved [30]. In addition to the manuscript, the convergence test is presented in the Supplementary Material. Therefore, for one rigorous coupled-wave calculation, the software gives out 271 reflected complex number magnetic fields for *p*-polarization and 271 reflected complex number electric fields for s-polarization.

### 2.2. Microscope Back Focal Plane Simulation

The two linear polarizations included in this study were x-polarization and y-polarization, respectively, as depicted in Figure 3b–c. The x-polarization has the electric field component pointing towards the positive x-direction in the back focal plane (BFP), in other words, perpendicular to the grating stripes, whereas the y-polarization has the electric field direction pointing along the y-axis in the BFP and parallel to the grating stripes. This section describes the optical microscope simulation and the BFP calculation steps, including:

Step 1 specifies the NA of the objective lens and the coupling refractive index (*n*_0_) for the immersion oil and the glass substrate. In this study, two objective lenses were investigated: the 1.7NA with *n*_0_ of 1.78 and the 1.49NA with *n*_0_ of 1.52.

**Figure 4:**
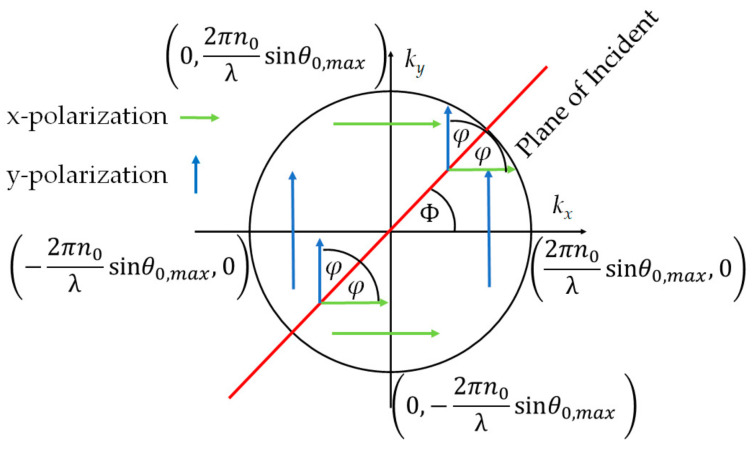
Shows x-polarization in green arrows and y-polarization in blue arrows and their wave vector space at the exit pupil function of the microscope objective.

Step 2 calculates the wave vector along the x, y, and z axes, *k_x_, k_y_*, and *k_z_*, respectively, at the exit pupil function of the objective lens, as depicted in Figure 4. The maximum *k_x_* and *k_y_* that an objective lens can provide are given by:

(1) $$k_{x,max},k_{y,max}=\frac{2\pi }{\lambda }NA=\frac{2\pi n_{0}}{\lambda }sin\theta_{0,max}$$

where $\theta_{0,max}$ is the maximum incident angle provided by the objective lens.

Step 3 determines the incident angle *θ*_0_ inside the glass substrate *n*_0_ for each (***k_x_***, ***k_y_***) coordinates in Figure 4 by calculating Equation (2).

(2) $$sin\theta_{0}=\sqrt{k_{x}^{2}+k_{y}^{2}}/\left(\frac{2\pi n_{0}}{\lambda }\right)$$

Step 4 is to work out the plane of incidence *ϕ* by Equation (3)

(3) $$\phi =tan^{-1}\left(k_{y}/k_{x}\right)$$

Step 5 is to work out the polarization angle *φ* as depicted in Figure 4b–c by Equations (4) and (5):

(4) φ=ϕ for the x-polarization

(5) $$\varphi =\phi +\frac{\pi }{2}\;\mathrm{for}\;\mathrm{the}\;y-\mathrm{polarization}$$

**Figure 5:**
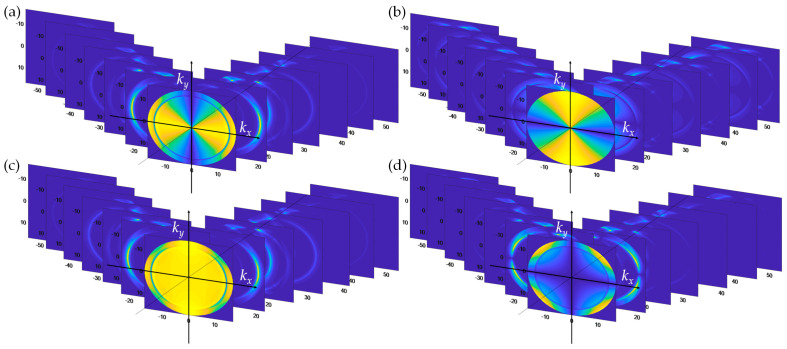
Shows diffracted BFPs (**a**) $H_{TM,m}\left(k_{x,m},k_{y}\right)$, (**b**) $E_{TE,m}\left(k_{x,m},k_{y}\right)$, (**c**) $E_{x,m}\left(k_{x,m},k_{y}\right)$, and (**d**) $E_{y,m}\left(k_{x,m},k_{y}\right)$.

Step 6 is to compute the optical responses using the rigorous coupled-wave analysis explained in the earlier section for each $\left(k_{x},k_{y}\right)$ coordinate pair in the BFP space. After the computation, these give out 271 diffracted reflected magnetic field BFPs $H_{TM,m}\left(k_{x,m},k_{y}\right)$ and 271 diffracted reflected electric field BFPs $E_{TE,m}\left(k_{x,m},k_{y}\right)$, as depicted in Figure 5a,b. Note that *m* is the *m*^th^ diffraction mode number and the $k_{x,m}$ is expressed by the Floquet equation [31], as shown in Equation (6).

(6) $$k_{x,m}=k_{x}+m\frac{2\pi }{\lambda_{g}}$$

Step 7 is to resolve the TM and TE components back to the $\left(k_{x},\;k_{y}\right)$ coordinate using Equations (7) and (8) as shown in Figure 5c,d:

(7) $$E_{x,m}\left(k_{x,m},k_{y}\right)=iH_{TM,m}\left(k_{x,m},k_{y}\right)cos\;\phi_{m}/n_{0}-E_{TE,m}\left(k_{x,m},k_{y}\right)sin\;\phi_{m}$$

(8) $$E_{y,m}\left(k_{x,m},k_{y}\right)=iH_{TM,m}\left(k_{x,m},k_{y}\right)sin\;\phi_{m}/n_{0}+E_{TE,m}\left(k_{x,m},k_{y}\right)cos\;\phi_{m}$$

where $\phi_{m}$ is the plane of incidence of the *m*^th^ reflected diffracted order calculated using Equation (9).

(9) $$\phi_{m}=tan^{-1}\left(k_{y}/k_{x,m}\right)$$

**Figure 6:**
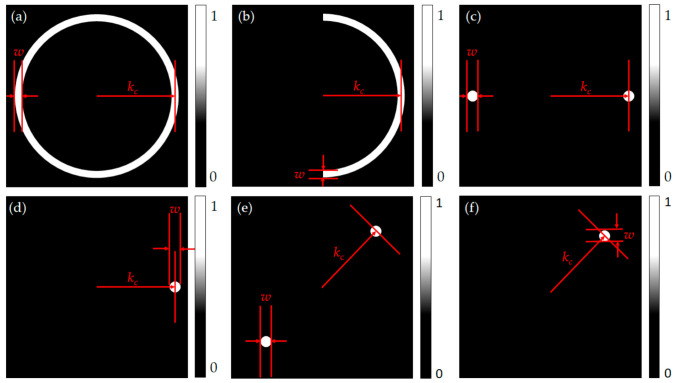
Shows the (**a**) annulus BFP aperture, (**b**) half annulus BFP aperture, (**c**) double-point BFP aperture, (**d**) single-point BFP aperture, (**e**) double-point BFP aperture with the azimuthal angle of 45°, and (**f**) single-point BFP aperture with the azimuthal angle of 45°.

Step 8 is to multiply the $E_{x,m}\left(k_{x,m},k_{y}\right)$ and the $E_{y,m}\left(k_{x,m},k_{y}\right)$ by amplitude pupil function $P\left(k_{x},k_{y}\right)\;$ considered each SPR microscope detection scheme as shown in Figure 6. Thus, there are six types of non-interferometric SPR microscopes in the scope of this study.

(1) Annulus BFP aperture reported by Tan et al. [24]; here, the responses of the microscope were simulated by defining the annulus aperture for the incident beam using two parameters, which were the center position of the aperture $k_{c}$, and the width of the aperture *w*, as depicted in Figure 6a.
(2) Single-sided annulus aperture; this is a modified version of the annulus aperture. Here, this left side of the aperture was blocked to study the effect of asymmetrical illumination, as shown in Figure 6b.
(3) Double point BFP illumination; Huang et al. [25] have proposed an SPR microscope, where the BFP is illuminated with a focal spot on one side along the *k_x_* axis. Here, the microscope proposed by Huang et al. is modified to illuminate both sides of the objective lens to investigate whether the symmetrical illumination can enhance the imaging performance. The position of the BFP illumination pupil function was defined by two parameters, which were the center position of the aperture $k_{c}$, and the aperture width *w*, as shown in Figure 6c. We also investigated the effect of the azimuthal angle by simulating the double point aperture with the azimuthal angle of 45 degrees, as depicted in Figure 6e.
(4) Single point BFP illumination; this is the SPR microscope proposed by Huang et al. [25]. The size of the BFP illumination was defined by the same parameters described in the double-point BFP as shown in Figure 6e,d. In addition, like the double point illumination, the 45 degrees azimuthal angle for the single point aperture was also investigated, as shown in Figure 6f.

**Figure 7:**
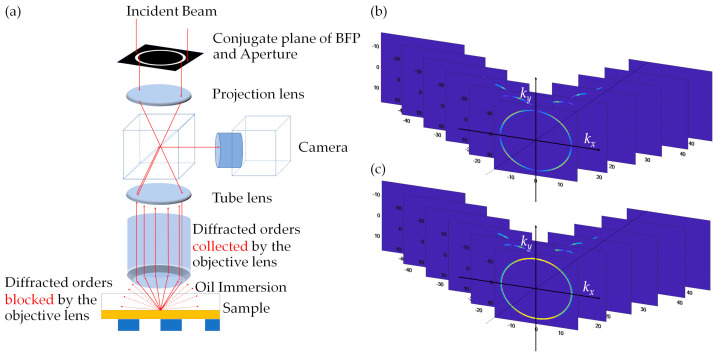
Shows the (**a**) diffractions collected and blocked by the objective lens, (**b**) diffracted BFPs $P\left(k_{x},k_{y}\right)E_{x,m}\left(k_{x,m},k_{y}\right)$, and (**c**) $P\left(k_{x},k_{y}\right)E_{y,m}\left(k_{x,m},k_{y}\right)$.

Step 9 Takes into account the objective lens NA allowing only the $P\left(k_{x},k_{y}\right)E_{x,m}\left(k_{x,m},k_{y}\right)$ and the $P\left(k_{x},k_{y}\right)E_{y,m}\left(k_{x,m},k_{y}\right)$ reflections with their diffraction angles within the range of angles that the objective lens NA can accommodate to be collected by the objective lens. On the other hand, the diffraction angles bigger than the capability of the objective lens missed the objective lens and did not contribute to the image formation, as the illumination part and the imaging part was separated, as depicted in Figure 7a. $P\left(k_{x},k_{y}\right)E_{x,m}\left(k_{x,m},k_{y}\right)$ and $\;P\left(k_{x},k_{y}\right)E_{y,m}\left(k_{x,m},k_{y}\right)$ reflections passing through the objective lens are depicted in Figure 7b,c.

Step 10 calculates the image responses corresponding to each $(k_{x},k_{y}$) coordinates in the BFP using Equation (10).

(10) $$I\left(k_{x},k_{y}\right)={\left|\;\overset{m=135}{\underset{m=-135}{\int }}P\left(k_{x},k_{y}\right)E_{x,m}\left(k_{x,m},k_{y}\right)e^{i\left(k_{x}-k_{x,m}\right)x}dm\right|}^{2}+{\left|\;\overset{m=135}{\underset{m=-135}{\int }}P\left(k_{x},k_{y}\right)E_{y,m}\left(k_{x,m},k_{y}\right)e^{i\left(k_{x}-k_{x,m}\right)x}dm\right|}^{2}$$

Step 11 sums all the intensity images corresponding to each ($k_{x},k_{y})$ coordinates for a non-interferometric response using Equation (11).

(11) $$I=\overset{k_{y,max}}{\underset{-k_{y,max}}{\int }}\overset{k_{x,max}}{\underset{-k_{x,max}}{\int }}I\left(k_{x},k_{y}\right)dk_{x}dk_{x}$$

### 2.3. Comparative Performance Parameters

**Figure 8:**
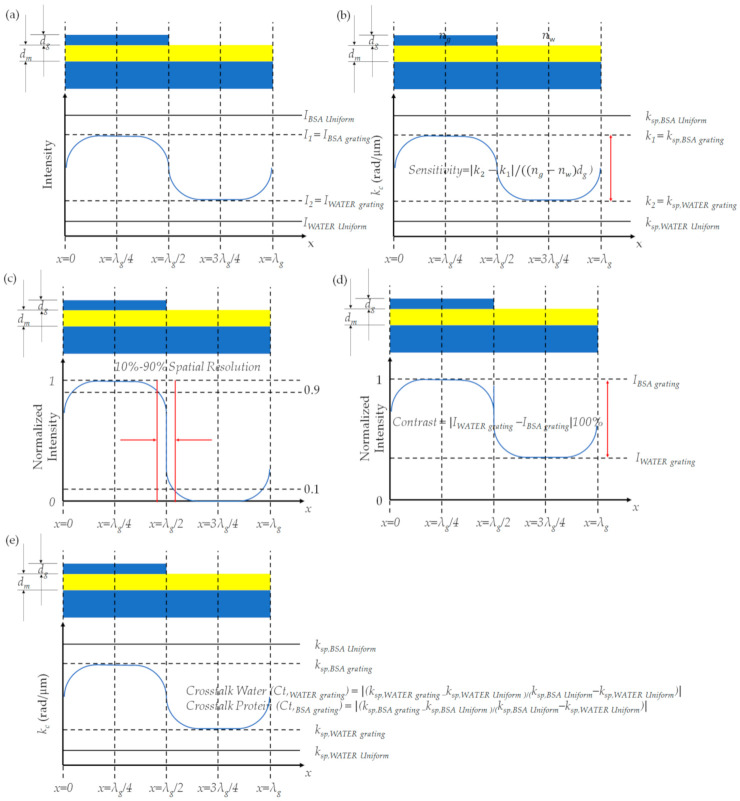
Shows the comparative performance parameters calculation details of the (**a**) intensity image from non-quantitative imaging, (**b**) recovered plasmonic angle for quantitative imaging and sensitivity calculation, (**c**) contrast calculation, (**d**) image normalization and 10–90% spatial resolution calculation, and (**e**) crosstalk calculation.

Computed images for the SPR microscopes for the non-quantitative imaging, as shown in Figure 8a, and the quantitative imaging shown in Figure 8b, were quantified using the following performance parameters.

(1) 10–90% spatial resolution (${\mathrm{Res}}_{10-90\%}$) defined as the transition length of the normalized image between the intensity of 0.1 to 0.9, as shown in Figure 8c.
(2) Contrast (***C***) is the absolute difference of image intensities divided by its maximum line scan intensity at the centers of the two grating materials, which is expressed in Equation (12) as depicted in Figure 8d.
  (12) $$C=100\%\times \left|I_{WATER\;grating}-I_{BSA\;grating}\right|$$
  where $I_{WATER\;grating}$ and $I_{BSA\;grating}$ are the normalized image intensities by dividing the intensity by the maximum intensity value of the line scan image for the centers of the two grating materials.
(3) Sensitivity (*S*) in the SPR measurement is defined as the change in surface plasmon wave-vector at the two centers of the grating $k_{sp,BSA\;grating}-k_{sp,WATER\;grating}\;$ over the change in sample refractive index and sample thickness product $(n_{g}-n_{w})d_{g}$. The S for the quantitative measurement is given by Equation (13) and depicted in Figure 8b.

(13) $$S=\frac{k_{sp,BSA\;Arating}-k_{sp,WATER\;grating}}{(n_{g}-n_{w})d_{g}}$$

(4) The relative value of plasmonic angles; it will be shown later that the recovered plasmonic angle for each microscope gives a slightly different absolute value of plasmonic angles. Therefore, the plasmonic angle will be calculated as relative values given by Equations (14)–(16) to compare across different configurations. The absolute value of a plasmonic angle; is the value of the plasmonic angle recovered in each microscope configuration. Note that the plasmonic angles $k_{sp,BSA\;grating}$ and $k_{sp,WATER\;grating}\;$, the plasmonic angles at the two centers at the x of 3*λ_g_*/4 for the water region and the  x of *λ_g_*/4 for the BSA region of the grating are depicted in Figure 8d. The $k_{sp,BSA\;uniform}$ and $k_{sp,WATER\;uniform}\;$ are the plasmonic angles measured for the uniform layer of the BSA protein and the bare gold sensor, respectively.

(14) $$\alpha_{sp,x\;grating}=\left|\frac{k_{sp,x\;grating}-k_{sp,WATER\;uniform}}{k_{sp,\;BSA\;uniform}-k_{sp,WATER\;uniform}}\right|$$

(15) $$\alpha_{sp,WATER\;uniform}=\left|\frac{k_{sp,WATER\;uniform}-k_{sp,WATER\;uniform}}{k_{sp,\;BSA\;uniform}-k_{sp,WATER\;uniform}}\right|=0\;$$

(16) $$\alpha_{sp,BSA\;uniform}=\left|\frac{k_{sp,\;BSA\;uniform}-k_{sp,WATER\;uniform}}{k_{sp,\;BSA\;uniform}-k_{sp,WATER\;uniform}}\right|=1$$

(5) Crosstalk (***Ct***) is defined as the deviation from the absolute plasmonic angles, as described by Equation (17) and depicted in Figure 8e.
  (17) $$Ct_{,water\;grating}=\left|\frac{k_{sp,WATER\;grating}-k_{sp,WATER\;uniform}}{k_{sp,\;BSA\;uniform}-k_{sp,WATER\;uniform}}\right|$$
  (18) $$Ct_{,BSA\;grating}=\left|\frac{k_{sp,BSA\;grating}-k_{sp,WATER\;uniform}}{k_{sp,\;BSA\;uniform}-k_{sp,WATER\;uniform}}\right|$$
  where $Ct_{,water\;grating}$ and $Ct_{,BSA\;grating}$ are the measurement crosstalk at the center of the water region and the center of the BSA region in the grating sample, respectively.

## 3. Results

### 3.1. Effects of Aperture Size and Position

The BFP line scans for the two uniform cases for 1.7NA are shown in Figure 1c. The full width at half maximum (*FWHM*) of the two plasmonic dips is 0.57 rad/μm. The FWHM is needed to quantify the effect of the annulus aperture and the circular aperture, measure the size of the aperture relative to the FWHM of the SPR dips so that this study can be applied to other plasmonic metals [32] or metamaterial surfaces [33] with a narrower or a broader dip.

**Figure 9:**
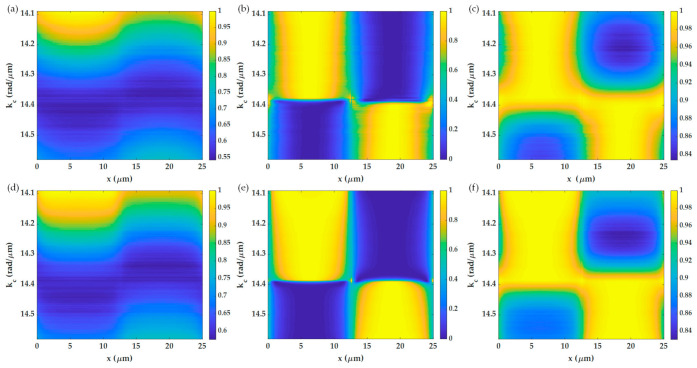
Shows line scans images at different annulus radius *k_c_* varying from 14.1 rad/μm to 14.6 rad/μm and the *w* of 0.06 rad/μm, equivalent to 0.1*FWHM* of SPR dip. The sample was the 25 μm grating period under the 1.7NA objective lens. (**a**) non-quantitative images of the grating for the x-polarization, (**b**) normalized image to the intensity range 0 to 1 for the x-polarization, (**c**) image contrast for the x-polarization, (**d**) non-quantitative images of the grating for the y-polarization, (**e**) normalized image to the intensity range 0 to 1 for the y-polarization, and (**f**) image contrast for the y-polarization.

Figure 9a,d shows line scans images at different annulus radius *k_c_* varying from 14.1 rad/μm to 14.6 rad/μm and the *w* of 0.06 rad/μm, equivalent to 0.1*FWHM* of SPR dip when the sample was the 25 μm grating period under the 1.7NA objective lens for the x-polarization and the y-polarization, respectively. It will be shown later that the angular scanning or *k_c_* scanning allows us to determine the local plasmonic angles of each position on the grating, enabling the quantitative imaging mode of the SPR microscopes.

**Figure 10:**
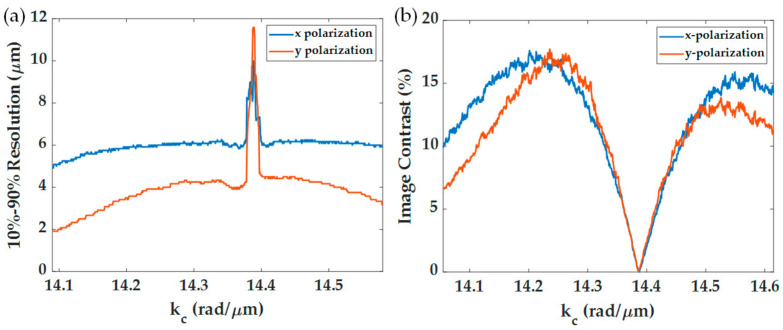
(**a**) 10–90% spatial resolution for x-polarization and y-polarization calculated from Figure 9b–e, and (**b**) image contrast in percentage for x-polarization and y-polarization calculated from Figure 9c–f using Equation (12). Note that the x-polarization is shown in blue curves, and the y-polarization is shown in red curves.

Each line scan image in Figure 9a,d can then be normalized for the 0 to 1 range to quantify each *k_c_* position’s spatial resolution imaging capability as shown in Figure 9b,e. The y-polarization has a shaper edge transition compared to the x-polarization. Figure 9c,f shows the image contrasts of the two polarization. Figure 10a shows the 10–90% spatial resolution for the x-polarization and the y-polarization calculated from Figure 9b,e, which were around 6.08 μm and 4.25 μm for x-polarization and y-polarization at *k_c_* of 14.3 rad/μm, respectively. The performance was much worse than the capability of the 1.7NA objective lens. The expected spatial resolution based on the Rayleigh criteria is 1.22λ/NA, which is 0.45 μm and 0.52 μm for the 1.7NA and 1.49NA objective lenses. The performance of the SPR microscope does not depend on the NA of the objective lens but rather the propagation length of the surface wave. Figure 10b shows the image contrast for different *k_c_* positions of the two polarizations; at a *k_c_* of 14.38 rad/μm, the images had no contrast and poor spatial resolution since the plasmonic dips of the two regions had the same intensity level as pointed out in Figure 1c. The maximum contrasts of 17% appeared when *k_c_* was at 0.15 rad/μm or 0.24 *FWHM* away from 14.38 rad/μm, corresponding to 14.25 rad/μm and 14.55 rad/μm. Note that the noise artifacts in Figure 9 and Figure 10 were from the quantization error of the scanning annulus aperture in the BFP. The BFP was computed by 651 pixels × 651 pixels representing the wave vector space of −16.8743 rad/μm to 16.8743 rad/μm. The *w* size of 0.06 rad/μm was chosen in this study, corresponding to 2.3 pixels. The quantization of scanning aperture in the BFP using caused the numerical noise.

**Figure 11:**
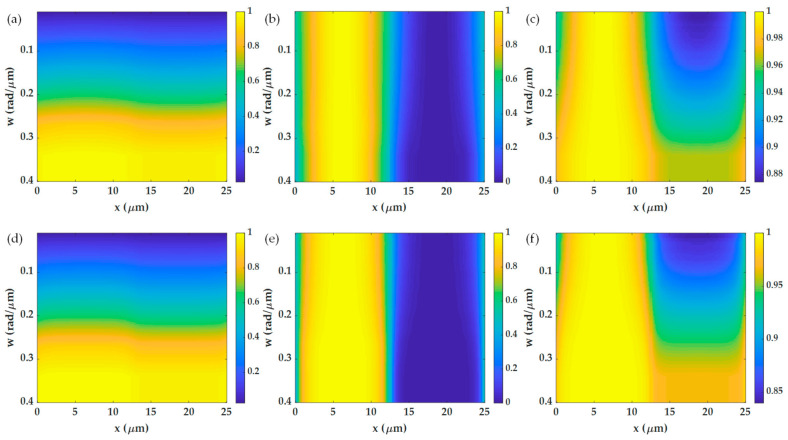
Shows image responses for non-quantitative imaging varying *w* from 0.01 rad/μm to 0.4 rad/μm and the *k_c_* of 14.3 rad/μm for (**a**) non-quantitative images of the grating for the x-polarization, (**b**) normalized image to the intensity range 0 to 1 for the x-polarization, (**c**) image contrast for the x-polarization, (**d**) non-quantitative images of the grating for the y-polarization, (**e**) normalized image to the intensity range 0 to 1 for the y-polarization, and (**f**) image contrast for the y-polarization.

**Figure 12:**
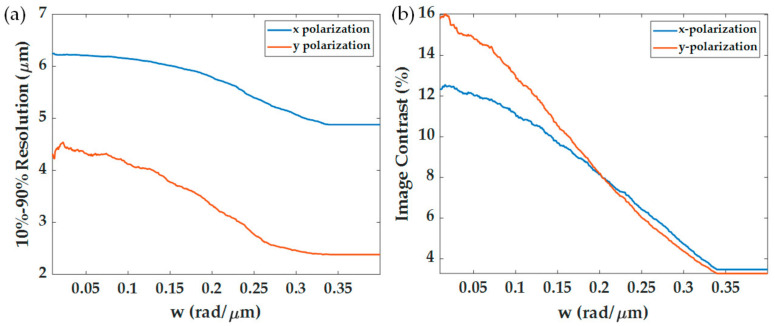
(**a**) 10–90% spatial resolution for x-polarization and y-polarization calculated from Figure 11b–e, and (**b**) image contrast in percentage for x-polarization and y-polarization calculated from Figure 11c–f using Equation (12). Note that the x-polarization is shown in blue curves, and the y-polarization is shown in red curves.

Figure 11 shows the effects of different aperture sizes *w* ranging from 0.01 rad/μm (0.018 *FWHM*) to 0.4 rad/μm (0.7 *FWHM*), and the *k_c_* of 14.3 rad/μm on the SPR imaging performance. Here the 1.7NA and 25-micron period grating were analyzed. Figure 11a,d shows that the wider aperture size can increase the optical intensity of the images for both the x-polarization and the y-polarization. Figure 11b,e shows normalized intensity images from the range of 0 to 1 to calculate the 10–90% spatial resolution; the bigger aperture size gives better spatial resolution, as shown in Figure 12a. Figure 11c,f shows normalization by dividing the line scan images with their maximum intensity to calculate image contrast for the x-polarization and the y-polarization, respectively. Figure 12b shows the contrasts for different aperture sizes; the bigger aperture degrades the image’s contrast. Thus, there is a tradeoff between spatial resolution and image contrast [15,34]. However, if the aperture becomes too large *w* of 0.35 rad/μm (0.61 *FWHM*), although the spatial resolution was better than the narrower apertures, the contrast became too low for imaging.

### 3.2. Effects of NA and propagation length of the SPs

It is well established that metals with higher attenuation, like aluminum [35], provide better spatial resolution at the expense of contrast between the grating regions. Here, we demonstrate the point by simulating the same grating period of 25 μm with *d_m_* of 35 nm instead of 50 nm and using a lower NA objective lens of 1.49 with the coupling index of immersion oil and the glass substrate *n_0_* of 1.52. We have explained the loss mechanism of the plasmonic material layer through coupling loss and ohmic loss in Pechprasarn et al. [36], that the thinner gold of 35 nm has a more substantial coupling loss; therefore, it has a shorter propagation length compared to the 50 nm gold. Table 1 shows the 10–90% spatial resolution and the contrast for different cases of NAs, thicknesses of gold, and the two polarizations at the *k_c_* of 14.3 rad/μm and *w* of 0.06 rad/μm. The resolution depends on the propagation length of the SP and the polarization direction, not the NA of the objective lens.

### 3.3. Imaging Performance Comparison for Different Microscope Configurations

**Figure 13:**
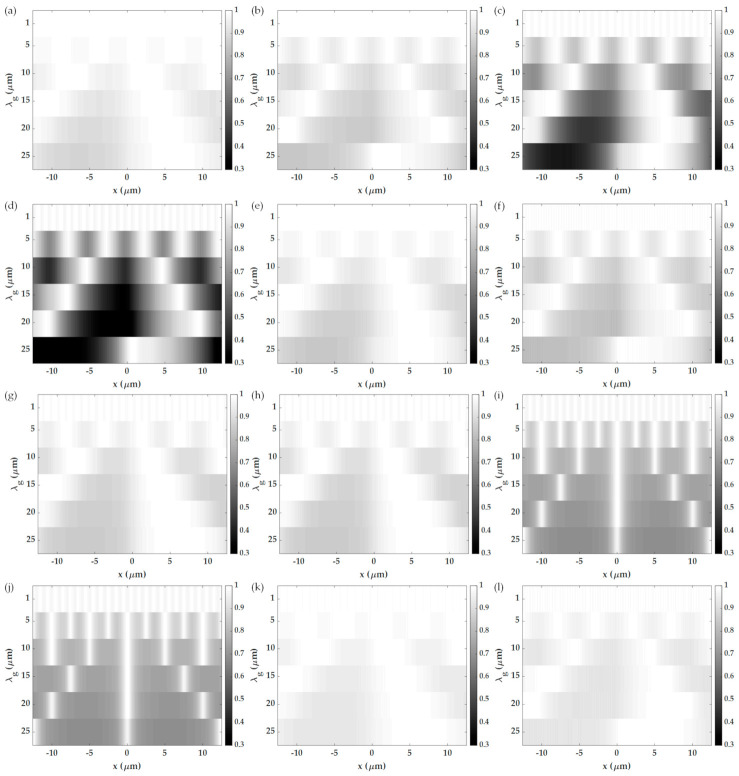
Images calculated for non-quantitative imaging for *w* of 0.06 rad/μm (0.1FWHM), *k_c_* of 14.3 rad/μm, different grating periods, and six types of apertures in this study: (**a**) annulus aperture for the x-polarization, (**b**) half annulus aperture covering the plasmonic dip for the x-polarization, (**c**) double point aperture for the x-polarization, (**d**) single point aperture for the x-polarization, (**e**) double point aperture with 45 degrees azimuthal angle for the x-polarization, (**f**) single point aperture with 45 degrees azimuthal angle for the x-polarization, (**g**) annulus aperture for the y-polarization, (**h**) half annulus aperture covering the plasmonic dip for the y-polarization, (**i**) double point aperture for the y-polarization, (**j**) single point aperture for the y-polarization, (**k**) double point aperture with 45 degrees azimuthal angle for the y-polarization, and (**l**) single point aperture with 45 degrees azimuthal angle for the y-polarization.

For non-quantitative imaging, Figure 13a–f shows images calculated for different BFP apertures. The *w* of 0.06 rad/μm (0.1 *FWHM*) and *k_c_* of 14.3 rad/μm have been employed to characterize the performance of the six microscope configurations, which are (1) annulus aperture [24], (2) half annulus aperture covering the plasmonic dip, (3) double-point aperture, (4) single point aperture [25], (5) double-point aperture with 45 degrees azimuthal angle, and (6) single-point aperture with 45 degrees azimuthal angle.

Figure 13a–f shows images calculated using the BFP apertures and different grating periods for the x-polarization, and Figure 13g–l is for the y-polarization. Table 2 summarizes the imaging performance parameters extracted from Figure 13. The y-polarization generally had better spatial resolution and contrast than the x-polarization. It is crucial to point out that although the y-polarization gave a superior performance to the x-polarization, the double-point aperture and the single point aperture at 0-degree azimuthal angle cannot form the correct grating image due to the diffracted orders were in the orthogonal direction to the plasmonic angles. For the unsymmetrical apertures, the half annulus aperture and the single point aperture produced images with unsymmetrical images causing an image shadowing effect, as reported in Huang et al. [25]. The spatial resolution depended on the grating period of the sample. There is a strong dependence between the spatial resolution and the contrast of all the non-interferometric SPR microscopes. The annuls aperture with the y-polarization provides the best spatial resolution performance compared to the other apertures without degrading the contrast.

**Figure 14:**
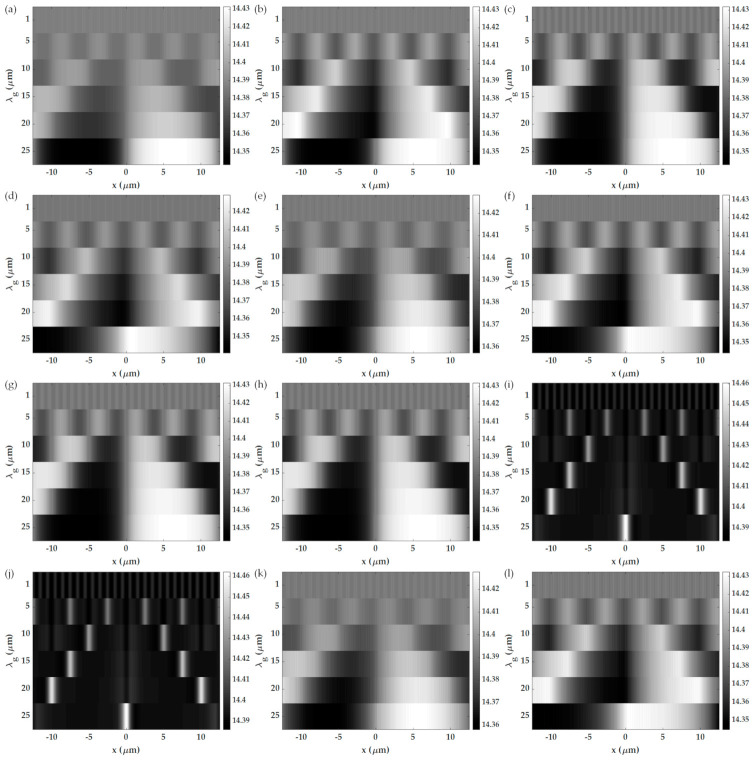
Recovered plasmonic wave vector *k_sp_* in rad/μm calculated for quantitative imaging for *w* of 0.06 rad/μm (0.1FWHM), different grating periods, and the six types of apertures in this study: (**a**) annulus aperture for the x-polarization, (**b**) half annulus aperture covering the plasmonic dip for the x-polarization, (**c**) double point aperture for the x-polarization, (**d**) single point aperture for the x-polarization, (**e**) double point aperture with 45 degrees azimuthal angle for the x-polarization, (**f**) single point aperture with 45 degrees azimuthal angle for the x-polarization, (**g**) annulus aperture for the y-polarization, (**h**) half annulus aperture covering the plasmonic dip for the y-polarization, (**i**) double point aperture for the y-polarization, (**j**) single point aperture for the y-polarization, (**k**) double point aperture with 45 degrees azimuthal angle for the y-polarization, and (**l**) single point aperture with 45 degrees azimuthal angle for the y-polarization.

Quantitative imaging can be achieved by scanning the center of the aperture positions *k_c_* and determine the *k_c_* position that gives the minimum intensity or the plasmonic wave vector positions *k_sp_*. Figure 14a–f shows the recovered plasmonic wave vector positions calculated for different BFP aperture types and different grating periods for the x-polarization, and Figure 14g–l is for the y-polarization. Table 3 summarizes the imaging performance parameters extracted from Figure 14. Like the non-quantitative imaging mode, the imaging performance parameters of the y-polarization are generally better than the x-polarization. The half annulus aperture provided the highest sensitivity of 127.058 rad/μm^2^ RIU^−1^ compared to the other apertures, followed by the full annulus aperture with the slightly lower sensitivity of 126.318 rad/μm^2^ RIU^−1^. There is also a tradeoff between sensitivity and spatial resolution. The single point aperture and the double point aperture cannot form a correct grating image because the polarization was in the orthogonal direction to the diffraction orders. The unsymmetrical apertures also suffered from an unsymmetrical image like the non-quantitative imaging. The annulus aperture and half annulus aperture can provide the optimal resolution, sensitivity, and crosstalk compared to the other aperture types. A grating period of at least 25 μm is required to measure low crosstalk plasmonic angles. Therefore, for SPR measurements, it is recommended from this research that the microfluidic channels should have the separation of at least 25 μm, and a full annulus aperture is in use for quantitative imaging.

## 4. Conclusions

This paper has demonstrated and explained a detailed procedure for simulating surface plasmon resonance optical microscopes using rigorous coupled-wave analysis and the back focal plane simulation. In addition, the theoretical framework to quantify and analyze six types of non-interferometric microscopes for non-quantitative and quantitative plasmonic imaging modes has been proposed and discussed, including (1) annulus aperture scanning, (2) half annulus aperture scanning, (3) single-point scanning, (4) double point scanning, (5) single-point scanning at 45 degrees azimuthal angle, and (6) double point scanning at 45 degrees azimuthal angle. For the two imaging modes, the y-polarization had better imaging performance than the x-polarization. However, the single-point scanning and the double-point scanning at 0 degrees azimuthal angle could not form a completed grating image. For non-quantitative imaging, there is a clear tradeoff between image contrast and spatial resolution. The resolution of the SPR microscopes depends on the propagation length of the SP, not the NA of the objective. For quantitative imaging, there is a tradeoff between spatial resolution and SPR sensitivity. From the findings, the complete annulus and the half annulus apertures with the sensing area separation of at least 25 µm are recommended for accurate quantitative plasmonic angle measurement.

## Figures and Tables

**Table 1:** Shows the 10–90% spatial resolutions and the image contrasts of 1.49NA and 1.7NA objective lenses, two gold thicknesses *d_m_* of 35 nm and 50 nm for the x-polarization, and the y-polarization; the *k_c_* of 14.3 rad/μm and *w* of 0.06 rad/μm.

Cases	$Res_{10-90\%},\;\mathrm{in}\;\mu m\;$	Contrast (%)
1.49NA, *d_m_* of 35 nm, x-polarization	5.50	3.270
1.49NA, *d_m_* of 50 nm, x-polarization	6.08	6.133
1.7NA, *d_m_* of 35 nm, x-polarization	3.58	5.961
1.7NA, *d_m_* of 50 nm, x-polarization	6.08	12.161
1.49NA, *d_m_* of 35 nm, y-polarization	6.50	2.137
1.49NA, *d_m_* of 50 nm, y-polarization	4.41	7.768
1.7NA, *d_m_* of 35 nm, y-polarization	2.25	6.182
1.7NA, *d_m_* of 50 nm, y-polarization	4.25	14.214

**Table 2:** The local gradient resolution and the image contrast for non-quantitative imaging mode extracted from the results in Figure 1.

Non-Quantitative Imaging													
**Method**	**Data**	**x-Polarization**						**y-Polarization**					
		**Grating Periods (** **μm** **)**						**Grating Periods (** **μm** **)**					
		**1**	**5**	**10**	**15**	**20**	**25**	**1**	**5**	**10**	**15**	**20**	**25**
Annulus ring	Res_10–90%_ (μm)	0.33	1.18	2.27	3.95	5.20	6.08	0.24	1.52	2.40	3.15	3.73	4.25
	C (%)	0.67	1.72	4.45	7.71	10.28	12.16	1.22	5.16	9.76	12.10	13.42	14.21
Half ring	Res_10–90%_ (μm)	0.41	1.92	3.83	5.65	6.87	7.75	0.41	1.92	3.83	5.65	6.87	7.75
	C (%)	0.83	6.29	11.13	13.89	15.45	16.09	1.23	5.20	9.83	12.18	13.51	14.30
Double point	Res_10–90%_ (μm)	0.27	1.80	3.57	3.30	4.27	5.67	n/a	n/a	n/a	n/a	n/a	n/a
	C (%)	2.18	17.10	31.12	43.29	54.70	62.38	n/a	n/a	n/a	n/a	n/a	n/a
Single point	Res_10–90%_ (μm)	0.36	1.87	3.73	5.75	7.53	8.50	n/a	n/a	n/a	n/a	n/a	n/a
	C (%)	2.69	33.61	57.12	69.10	75.34	78.50	n/a	n/a	n/a	n/a	n/a	n/a
Single point 45°	Res_10–90%_ (μm)	0.33	1.80	2.40	3.95	4.93	5.92	0.39	1.78	2.30	4.05	5.07	6.08
	C (%)	0.52	3.29	7.16	11.22	14.02	15.78	1.47	3.15	6.97	11.01	13.80	15.58
Double point 45°	Res_10–90%_ (μm)	0.41	1.58	4.00	5.80	6.53	6.50	0.42	1.97	3.90	5.55	6.40	6.33
	C (%)	1.62	7.87	14.65	17.62	18.85	19.28	2.45	7.83	14.28	17.13	18.39	18.81

n/a = Not applicable.

**Table 3:** Shows the 10–90% spatial resolutions, image contrasts using Equation (12), sensitivity using Equation (13), and crosstalk using Equation (17) for quantitative imaging mode extracted from the plasmonic wave vector *k_sp_* in Figure 14.

Quantitative Imaging													
**Method**	**Data**	**x-Polarization**						**y-Polarization**					
		**Grating Periods (µ** **m** **)**						**Grating Periods (µ** **m** **)**					
		**1**	**5**	**10**	**15**	**20**	**25**	**1**	**5**	**10**	**15**	**20**	**25**
Annulus ring	Res_10–90%_ (μm)	0.28	1.28	2.00	4.00	5.07	6.08	0.19	1.52	2.40	3.15	3.73	4.25
	S (rad/μm^2^RIU^−1^)	1.12	13.82	29.68	54.11	75.48	92.92	7.04	39.40	79.96	103.49	117.60	126.32
	Ct protein	0.53	0.48	0.41	0.31	0.22	0.15	0.59	0.40	0.23	0.13	0.07	0.04
	Ct water	0.46	0.42	0.36	0.27	0.19	0.13	0.47	0.29	0.15	0.07	0.02	0.02
Half ring	Res_10–90%_ (μm)	0.39	1.70	3.60	5.40	6.40	7.08	0.19	1.52	2.40	3.15	3.73	4.25
	S (rad/μm^2^ RIU^−1^)	1.14	13.12	28.87	53.25	74.74	92.33	7.09	39.68	80.50	104.15	118.31	127.06
	Ct protein	0.53	0.48	0.42	0.32	0.23	0.15	0.58	0.40	0.23	0.12	0.06	0.03
	Ct water	0.47	0.42	0.36	0.27	0.19	0.13	0.48	0.30	0.15	0.07	0.02	0.01
Double point	Res_10–90%_ (μm)	0.24	1.88	3.20	3.70	5.13	6.25	n/a	n/a	n/a	n/a	n/a	n/a
	S (rad/μm^2^ RIU^−1^)	1.72	8.75	18.55	39.34	59.54	79.53	n/a	n/a	n/a	n/a	n/a	n/a
	Ct protein	0.53	0.50	0.46	0.38	0.30	0.22	n/a	n/a	n/a	n/a	n/a	n/a
	Ct water	0.46	0.44	0.40	0.32	0.25	0.19	n/a	n/a	n/a	n/a	n/a	n/a
Single point	Res_10–90%_ (μm)	0.33	1.87	3.63	5.60	7.07	8.08	n/a	n/a	n/a	n/a	n/a	n/a
	S (rad/μm^2^RIU^−1^)	1.72	8.08	17.66	38.70	59.11	78.72	n/a	n/a	n/a	n/a	n/a	n/a
	Ct protein	0.53	0.50	0.47	0.39	0.30	0.22	n/a	n/a	n/a	n/a	n/a	n/a
	Ct water	0.46	0.44	0.40	0.33	0.26	0.19	n/a	n/a	n/a	n/a	n/a	n/a
Single point 45°	Res_10–90%_ (μm)	0.41	1.75	2.63	3.85	4.87	5.75	0.40	1.82	2.67	4.15	5.13	5.92
	S (rad/μm^2^ RIU^−1^)	3.57	15.58	35.85	61.52	84.95	99.88	1.23	7.80	29.47	57.66	82.19	98.58
	Ct protein	0.52	0.47	0.39	0.28	0.18	0.11	0.53	0.51	0.42	0.30	0.19	0.12
	Ct water	0.45	0.40	0.32	0.22	0.13	0.07	0.46	0.43	0.34	0.23	0.14	0.08
Double point 45°	Res_10–90%_ (μm)	0.42	1.63	3.77	5.30	6.47	6.67	0.41	1.75	3.67	5.30	6.20	6.50
	S (rad/μm^2^ RIU^−1^)	3.64	15.04	34.59	59.73	82.54	97.55	1.00	6.88	27.73	55.74	80.02	96.68
	Ct protein	0.52	0.47	0.39	0.29	0.18	0.12	0.53	0.50	0.42	0.30	0.19	0.12
	Ct water	0.45	0.41	0.33	0.23	0.14	0.09	0.46	0.44	0.36	0.25	0.16	0.09

n/a = Not applicable.

## Supplementary Materials

The following are available online at https://www.mdpi.com/article/10.3390/s21155230/s1, Figure S1: Shows RCWA simulation convergence tests using the incident wavelength λ of 633 nm; Figure S2. Shows RCWA simulation convergence tests using the incident wavelength λ of 633 nm.
