# Supplementary material for: Performance Analysis of Non-Interferometry Based Surface Plasmon Resonance Microscopes
Source: Sensors (Basel). 2021 Aug 2;21(15):5230. doi: 10.3390/s21155230 (PMC8347916; doi:10.3390/s21155230)
Supplement: Supplementary file 1 [file sensors-21-05230-s001.zip › sensors-1303899-supplementary.pdf]

Supplementary material

The convergence curves showing the stability in the zeroth-order reflectance as a function of the number of diffracted orders are shown in Figure S1 and Figure S2. The two figures show all the marginal cases and polarization directions in this study, including:

Figure S1a: λ=633nm, λ_g=1μm, θ_0=0 rad, ϕ=0 rad and ψ=0 rad

Figure S1b: λ=633nm, λ_g=25μm, θ_0=0 rad, ϕ=0 rad and ψ=0 rad

Figure S1c: λ=633nm, λ_g=1μm, θ_0=0 rad, ϕ=0 rad and ψ=π/2 rad

Figure S1d: λ=633nm, λ_g=25μm, θ_0=0 rad, ϕ=0 rad and ψ=π/2 rad

Figure S1e: λ=633nm, λ_g=1μm, θ_0=0 rad, ϕ= π/2 rad and ψ= 0 rad

Figure S1f: λ=633nm, λ_g=25μm, θ_0=0 rad, ϕ= π/2 rad and ψ= 0 rad

Figure S1g: λ=633nm, λ_g=1μm, θ_0=0 rad, ϕ= π/2 rad and ψ= π/2 rad

Figure S1h: λ=633nm, λ_g=25μm, θ_0=0 rad, ϕ= π/2 rad and ψ= π/2 rad

Figure S2a: λ=633nm, λ_g=1μm, θ_0=1.45 rad, ϕ=0 rad and ψ=0 rad

Figure S2b: λ=633nm, λ_g=25μm, θ_0=1.45 rad, ϕ=0 rad and ψ=0 rad

Figure S2c: λ=633nm, λ_g=1μm, θ_0=1.45 rad, ϕ=0 rad and ψ=π/2 rad

Figure S2d: λ=633nm, λ_g=25μm, θ_0=1.45 rad, ϕ=0 rad and ψ=π/2 rad

Figure S2e: λ=633nm, λ_g=1μm, θ_0=1.45 rad, ϕ= π/2 rad and ψ= 0 rad

Figure S2f: λ=633nm, λ_g=25μm, θ_0=1.45 rad, ϕ= π/2 rad and ψ= 0 rad

Figure S2g: λ=633nm, λ_g=1μm, θ_0=1.45 rad, ϕ= π/2 rad and ψ= π/2 rad

Figure S2h: λ=633nm, λ_g=25μm, θ_0=1.45 rad, ϕ= π/2 rad and ψ= π/2 rad

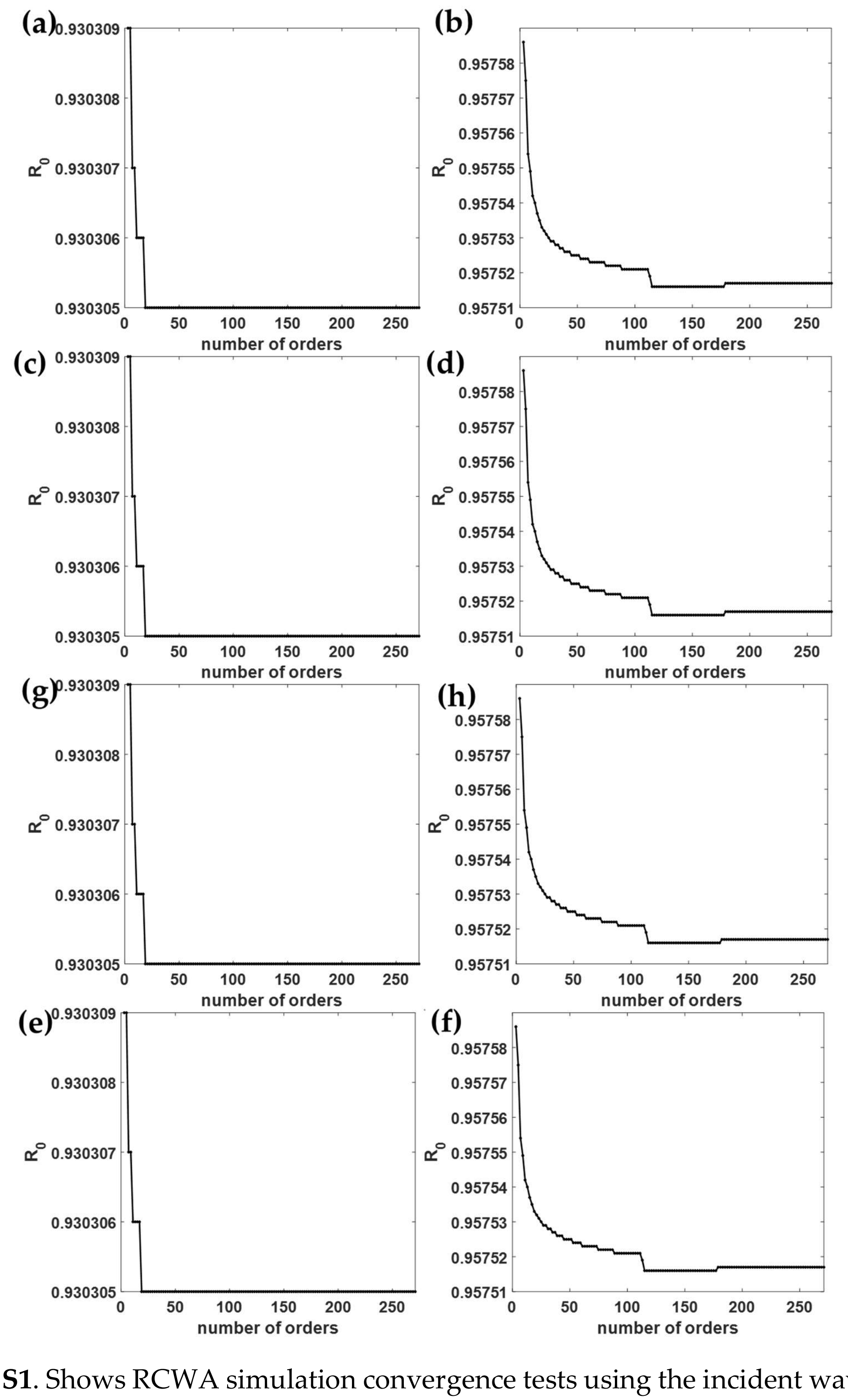

Figure S1. Shows RCWA simulation convergence tests using the incident wavelength λ of 633nm and **(a)** λ_g=1μm, θ_0=0 rad, ϕ=0 rad and ψ=0 rad. **(b)** λ_g=25μm, θ_0=0 rad, ϕ=0 rad and ψ=0 rad, **(c)** λ_g=1μm, θ_0=0 rad, ϕ=0 rad and ψ=π/2 rad, **(d)** λ_g=25μm, θ_0=0 rad, ϕ=0 rad and ψ=π/2 rad, **(e)** λ_g=1μm, θ_0=0 rad, ϕ= π/2 rad and ψ= 0 rad, **(f)** λ_g=25μm, θ_0=0 rad, ϕ= π/2 rad and ψ= 0 rad, **(g)** λ_g=1μm, θ_0=0 rad, ϕ= π/2 rad and ψ= π/2 rad, and **(h)** λ_g=25μm, θ_0=0 rad, ϕ= π/2 rad and ψ= π/2 rad

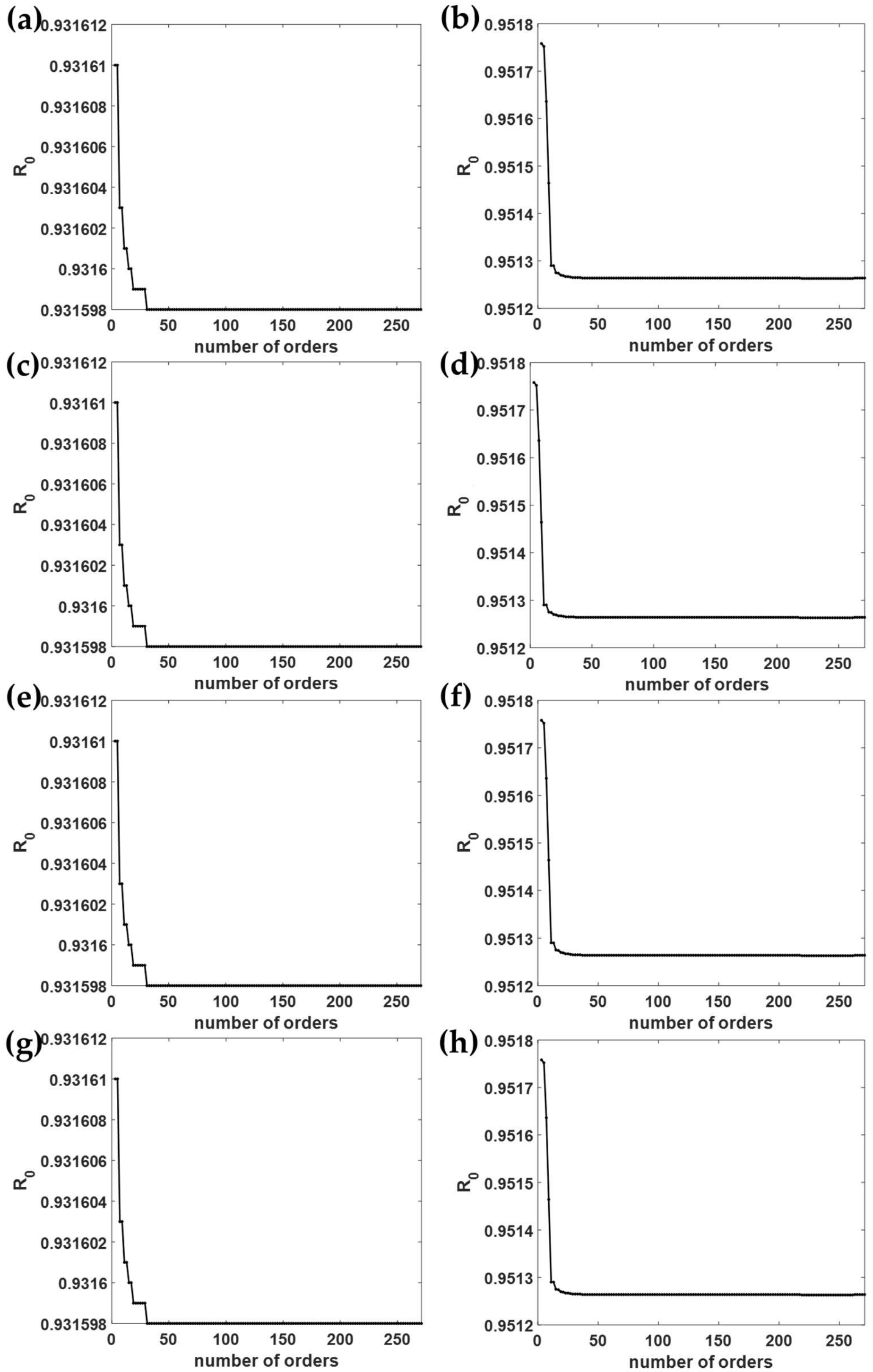

Figure S2. Shows RCWA simulation convergence tests using the incident wavelength λ of 633nm and **(a)** λ_g=1μm, θ_0=1.45 rad, ϕ=0 rad and ψ=0 rad. **(b)** λ_g=25μm, θ_0=1.45 rad, ϕ=0 rad and ψ=0 rad, **(c)** λ_g=1μm, θ_0=1.45 rad, ϕ=0 rad and $\psi=\pi/2$ rad, **(d)** λ_g=25μm, θ_0=1.45 rad, ϕ=0 rad and $\psi=\pi/2$ rad, **(e)** λ_g=1μm, θ_0=1.45 rad, $\phi= \pi/2$ rad and ψ= 0 rad, **(f)** λ_g=25μm, θ_0=1.45 rad, $\phi= \pi/2$ rad and ψ= 0 rad, **(g)** λ_g=1μm, θ_0=1.45 rad, $\phi= \pi/2$ rad and $\psi= \pi/2$ rad, and **(h)** λ_g=25μm, θ_0=1.45 rad, $\phi= \pi/2$ rad and $\psi= \pi/2$ rad

In conclusion, the 271 diffracted orders employed in the RCWA simulations can provide convergence for all the cases in the scope of the study.
